# Metabolomic study of human tissue and urine in clear cell renal carcinoma by LC-HRMS and PLS-DA

**DOI:** 10.1007/s00216-018-1059-x

**Published:** 2018-04-16

**Authors:** Joanna Nizioł, Vincent Bonifay, Krzysztof Ossoliński, Tadeusz Ossoliński, Anna Ossolińska, Jan Sunner, Iwona Beech, Adrian Arendowski, Tomasz Ruman

**Affiliations:** 1 Faculty of Chemistry, Rzeszow University of Technology, 35-959 Rzeszow, Poland; 2 Department of Microbiology and Plant Biology, University of Oklahoma, Norman, OK 73019 USA; 3 Department of General Surgery and Urology, John Paul II Hospital, Grunwaldzka 4 St., 36-100 Kolbuszowa, Poland; 4 Department of Chemistry, Montana State University, 103 Chemistry and Biochemistry Building, Bozeman, MT 59717 USA; 5 Center of Biofilm Engineering, Montana State University, 366 Barnard Hall, Bozeman, MT 59717 USA

**Keywords:** Renal cell carcinoma, Mass spectrometry, Biomarker, Cancer biomarker, Kidney

## Abstract

Renal cell carcinoma (RCC) is the most prevalent and lethal malignancy of the kidney. Despite all the efforts made, no tissue biomarker is currently used in the clinical management of patients with kidney cancer. A search for possible biomarkers in urine for clear cell renal cell carcinoma (ccRCC) has been conducted. Non-targeted metabolomic analyses were performed on paired samples of surgically removed renal cancer and normal tissue, as well as on urine samples. Extracts were analyzed by liquid chromatography/high-resolution mass spectrometry (LC-HRMS). Hydroxybutyrylcarnitine, decanoylcarnitine, propanoylcarnitine, carnitine, dodecanoylcarnitine, and norepinephrine sulfate were found in much higher concentrations in both cancer tissues (compared with the paired normal tissue) and in urine of cancer patients (compared with control urine). In contrast, riboflavin and acetylaspartylglutamate (NAAG) were present at significantly higher concentrations both in normal kidney tissue as well as in urine samples of healthy persons. This preliminary study resulted in the identification of several compounds that may be considered potential clear cell renal carcinoma biomarkers.

**Electronic supplementary material:**

The online version of this article (10.1007/s00216-018-1059-x) contains supplementary material, which is available to authorized users.

**Graphical abstract:**
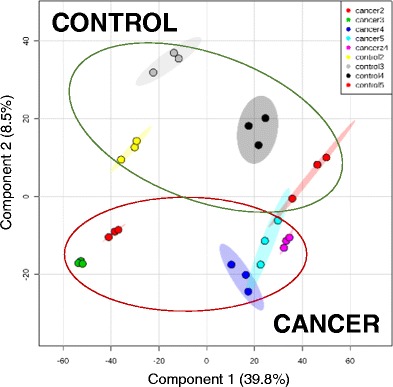
PLS-DA plot based on LC-MS data for normal and cancer human tissue samples. The aim of this work was the identification of up- and downregulated compounds that could potentially serve as renal cancer biomarkers.

## Introduction

Biomarkers provide a powerful approach to understanding diseases with applications in epidemiology, clinical trials, screening, diagnosis, and prognosis. Defined as alterations in the constituents of tissues or body fluids, they often offer the means for classification of a disease and can extend our knowledge about the underlying pathogenesis of disease. Theoretically, efficient biomarkers can also reflect the entire spectrum of disease from the earliest manifestation to the terminal stage. The development of cancer therapies is increasingly dependent on the understanding of tumor biology, and biomarkers are becoming essential tools in the field of medicine.

Renal cell carcinoma (RCC) is the most prevalent and lethal malignancy of the kidney, accounting for nearly 90% of all renal tumors and representing 2–3% of all adult malignant tumors [1, 2]. RCC is among the ten most common cancers worldwide and is the second most lethal urinary cancer after bladder. According to statistics published by GLOBOCAN in 2012, there were approximately 337,800 new cases of renal cancer and 143,400 kidney cancer-related deaths worldwide [3].

RCC is now thought to be a morphologically and genetically heterogeneous disease that can be classified into several different subtypes, such as clear cell RCC (ccRCC), papillary RCC, and chromophobe RCC [4, 5]. Clear cell RCC is the most prevalent histologic subtype of kidney cancer, accounting for more than 75% of all RCCs [6].

A favorable prognosis (95% survival after 5 years) can be achieved by radical nephrectomy with nephron-sparing surgery when kidney cancer is detected and treated at an early stage [7]. Unfortunately, most patients do not experience early warning signs, such as fever, fatigue, night sweats, or weight loss. Thus, as many as one third of patients are at an advanced stage of the disease and have metastatic tumors beyond the kidney, at the time of diagnosis. The lack of adequate therapies at this stage is usually associated with poor prognosis and long-term survival rates (5 years) [8–10]. Furthermore, RCC exhibits a high degree of intrinsic drug resistance and is, furthermore, highly resistant to radiation treatments [11]. This limits the treatment options and their effectiveness, although targeted therapies provide some survival benefit [12, 13].

Recent studies have renewed interest in the alterations in cellular metabolism associated with a range of diseases, including cancers, and it is now widely accepted that metabolomics can be a powerful tool not only for disease detection, diagnosis, as well as treatment guidance and assessment but also for the elucidation of the molecular processes behind the disease states [1, 2].

RCC is currently recognized as a metabolic disease [14] and was previously studied by the metabolomic analysis of body fluids, such as plasma [15], serum [16–18], and urine [19–23], as well as renal tissue [24]. Analytical methods used for RCC tissue metabolomic studies include ^1^H nuclear magnetic resonance (NMR) [25], gas chromatography/mass spectrometry (GC-MS) [21, 26, 27], liquid chromatography/mass spectrometry (LC-MS) [21, 24, 27–31], mass spectrometry using ambient ionization techniques, such as desorption electrospray ionization (DESI) [32, 33], and probe electrospray ionization (PESI) [34]. Metabolomics of RCC have been combined with other -omic approaches, such as transcriptomics [29, 30] and proteomics [24].

Metabolomic studies of RCC have been used not only for the identification of biomarkers [25, 28, 34], the differentiation of different phenotypes of RCC [26, 32], detection of metastases [25] but also to enhance understanding of the pathogenesis, progression of disease, assessment of the response to novel nonsurgical therapeutic strategies, and the early detection of recurrences [16, 35].

There is, thus, an increasing awareness of the promise of metabolomic characterization of kidney diseases, including RCC [24, 31, 36, 37]. There is potential for early detection, accurate diagnosis and staging, detection of metastasis, individualized treatments, prediction of patients’ outcome, and monitoring of response to treatment. However, comparative profiling of low molecular weight compounds, such as sugars, lipids and amino acids in cancer tissue, as contrasted with the corresponding normal tissue, is still a poorly explored area. Despite all the efforts made, there is still no agreement on clinically relevant tissue and biofluid-based biomarkers that could be used for the proper management of kidney cancer patients or on the analytical procedures to be used. This highlights the importance of continuous development and refinement of metabolomic strategies.

In the present study, metabolic profiling has been performed on both tissue and urine samples from patients with renal cancer. Cancer tissue was compared with healthy kidney tissue from the same patients, while urine from healthy subjects served as control for the urine samples from the cancer patients. Features that were significantly more or less abundant in either tissue or urine samples were identified. A number of potential biomarkers for renal cancer have been identified.

## Materials and methods

### Participants

Human kidney tissue and urine samples were obtained between June and September of 2015 from seven patients, who had kidney cancer and were scheduled for radical nephrectomy. Bioethics Committee at the University of Rzeszow (Poland) approved the study protocol. Specimens and clinical data from patients involved in the study were collected with written consent. Patients had been diagnosed with ccRCC. Four of the patients with ccRCC donated 1 cm^3^ of renal tissue removed ex vivo after radical surgical resection of kidney. These samples contained both cancerous and adjacent normal tissue. All patients donated 100 ml of urine each. Urine control samples were collected from 15 healthy volunteers, for which the presence of renal tumors had been excluded by abdominal ultrasound. For brevity, these samples are here referred to as “cancer urine” and “control urine,” respectively. Patient characteristics are provided in Table 1.

**Table 1:** Clinical characteristics of study group and controls

	ccRCC	Healthy urine donor
Total	4	15
Age (years)	57–82	41–78
Mean	68	58
Stage (T)		
T1	2	–
T2	1	–
T3	1	–
Nodes (N)		
N0	4	–
N1	0	–
Metastases (M)		
M0	4	–
M1	0	–
Grade (Fuhrman)		
II	2	–
III	2	–
Tumor dimension (largest, cm)		
Range	4.5–7.8	–
Mean	6	–

### Chemicals and reagents

Acetonitrile, tetrahydrofurane (THF), water, and formic acid were of HPLC-MS grade and purchased from Aldrich.

### Sample pretreatment

For tissue-based metabolomics, data obtained for the cancer tissue samples was compared with the data obtained for the normal tissue. The cancer and normal tissue samples for each patient were obtained from the same tissue specimen and were located 8–9 mm apart. All cancerous tissue samples were examined by uropathologists and graded according to both the Fuhrman and the American Joint Committee on Cancer clinical staging systems. Clinical characteristics of case and controls groups are given in Table 1). Normal and cancer tissue sections were cut out from central parts of RCC and normal tissue (∼ 1 mg from a ∼ 1-×-1-mm area), respectively, from each of the four specimens used in this study. Metabolites from each section were obtained by application of fast three times freezing/unfreezing temperature cycling with 100 μl of water (“water extracts”) or THF (“THF extracts”) and vortexed. Samples were then rapidly frozen and solvents removed by freeze-drying in speedvac-type equipment. Dried extracts were dispensed into 130 μl of LC-MS-grade water (for water-based analysis) or 1 ml of isopropyl alcohol (for THF-extraction analysis). The mixtures were vortexed for 30 s and centrifuged at 10,000×*g* for 1 min at ambient temperature, and the supernatant was transferred to an autosampler vial (2 ml) for LC-MS analysis.

For the urine-based study, data obtained for cancer patients as a group was compared with data for the control group of 15 healthy volunteers. Urine samples were collected and handled in a uniform manner to ensure consistency. Volumes of 100 μl of urine were diluted with 130 μl of LC-MS-grade water and subjected to vortexing, centrifugation, and supernatant collection as described above. Ten microliters of each aqueous solution were injected on Agilent UHPLC system. For each sample data, acquisition was performed in triplicate.

### Instrumentation

Liquid chromatography/high-resolution mass spectrometry (LC-HRMS) analyses were carried out using an Agilent 1290 ultra-high-performance liquid chromatograph (UHPLC) coupled to an Agilent 6538 quadrupole time-of-flight (QqTOF) mass spectrometer fitted with an electrospray ionization (ESI) source operated in positive ion mode (Agilent, Santa Clara, CA, USA).

LC separation was carried out using a SeQuant® ZIC®-HILIC column (5 μm, 150 × 4.6 mm, The Nest Group, Inc., Mass., USA) with a flow rate of 0.3 ml/min. A linear gradient was applied from 80 to 20% acetonitrile for the first 30 min, followed by 5% acetonitrile for an additional 8 min. The injection volume was 10 μl. Mass spectrometer parameters were as follows: ion-source gas temperature, 325 °C; capillary voltage, 4000 V; fragmentor voltage, 120 V; nebulizer pressure, 20 psi; sheath gas flow, 10 l/min; *m/z* range, 50–1100; data acquisition rate, 4 GHz; and 1.3 spectrum recorded/s. Approximately 130 authentic standards (mixture of amino acids, carbohydrates, energy metabolism metabolites, etc.) were used to calibrate the retention time calculator with any new column [38]. Before starting LC-MS measurements, 30 authentic standards were injected to validate the state of the column.

### Data processing

Raw MS data was processed using the IDEOM version 19 [39] workflow. This utilizes XCMS Centwave [40] for peak detection and mzMatch, R [41] for peak alignment between triplicates and between samples, for filtering and for the storage of the data in peak ML-formatted files. Feature alignment was performed with a retention time window of 30 s and a mass error window of 5 ppm. Scripts for XCMS [42] and mzMatch are coded in the R environment.

In the alignment procedure, peaks obtained in three different UHPLC-HRMS experiments (triplicate injections) are determined to be formed from the same compound, based on their appearance at nearly the same retention time and *m/z* value. Signals of isotopomers were identified and assigned to their respective quasi-molecular ion ([M + H]^+^ in positive ion mode). The monoisotopic mass of the corresponding neutral was obtained from that of the parent ion by subtracting the proton mass. The alignment procedure results in a list of “features,” each associated with a monoisotopic mass (for the neutral M), a retention time, and a total ion abundance. The calculated mass values for the neutral compounds, M, were used throughout the manuscript, instead of *m/z* for the MH^+^ ions. Unless the identification of a parent ion in a group of peaks as MH^+^ is erroneous, each feature will correspond to an actual compound. Alignment of detected peaks was performed separately for the set of samples extracted into THF and into water, respectively.

A major objective of this metabolomic study is to identify (putative) compounds that are over- or under-expressed in renal cancer as opposed to normal renal tissue. For features, the terms “over-abundant” and “under-abundant” were used, while “over-expressed” and “under-expressed” were used for (putatively) identified metabolites. Detailed LC-MS data discussed in this work is available in the Electronic supplementary material (ESM, Table S2).

Lists of detected features were matched against the IDEOM’s version of the Kyoto Encyclopedia of Genes and Genomes (KEGG) metabolite database [43] using a mass error tolerance of 4 ppm. Retention times for authentic standards, and a retention time prediction model, were included for ZIC-HILIC chromatography data [38]. For a putative identification, the maximum difference allowed between calculated and observed RT was 5% for authentic standards and 45% for other metabolites. Putative identifications were also guided by searches on the Madison-Qingdao Metabolomics Consortium Database (MMCD) [44] and the Human Metabolome Database (HMDB) [45].

Multivariate statistical analyses were performed using Metaboanalyst 3.0 [46].

## Results and discussion

Examples of total ion chromatograms for cancer and normal tissue extracts are shown in the ESM (Fig. S1). Similar comparison of chromatograms for cancer patient urine and control urine samples is shown in the ESM (Fig. S2). For water-extract-based analysis, a total of 4040 features were detected in the set of tissue samples and a total of 3368 in the set of urine samples. Each feature is associated with an exact mass, a retention time, and average abundancies in cancer and normal tissue, as well as with urine samples from patients with and without renal cancers. Results for cancer tissue as compared with normal tissue or in cancer urine as opposed to normal urine, are listed in Table 2.

**Table 2:** List of features that are over-abundant in either cancer (**1–4**, **14–20**) tissue or normal (**5–13**, **21–23**) tissue or over-abundant either in urine from cancer patients (**2**, **4**, **6**, **10**, **15–18**, **19–22**, **23–28**) or from control patients (**3**, **9**, **11**, **13**, **17**)

No.	Formula	Mass^a^	Mass error (ppm)	Retention time (min)	Putative metabolite	Average abundances			
						Tissue		Urine	
						Normal	Cancer	Control	Cancer
1	C_12_H_20_O_10_	324.1059	0.7	0.81	Bis-fructose 2′,1:2,1′-dianhydride	116	*34,973*	–	–
2	C_8_H_11_NO_6_S	249.0309	0.8	0.98	Norepinephrine sulfate	174	*999*	380	*630*
3	C_5_H_10_N_2_O_3_	146.0694	1.5	0.80	Glutamine	23,471	*31,944*	*15,542*	12,212
4	C_5_H_9_NO_4_	147.0532	0.2	0.94	Glutamate	170,025	*196,812*	1451	*1603*
5	C_10_H_21_NOS	203.1335	− 4.5	0.85	Methylthiononanaldoxime	*46,617*	6349	–	–
6	C_9_H_17_NO_3_	187.1156	− 0.2	2.52	*N*-Heptanoylglycine	*8801*	1133	513	*1021*
7	C_9_H_19_NO_2_	173.1418	1.5	3.27	Amino-nonanoic acid	*6276*	427	–	–
8	C_33_H_40_O_22_	788.2030	2.4	7.87	Quercetin sophoroside glucoside	*3509*	231	–	–
9	C_17_H_20_N_4_O_6_	376.1384	0.4	4.16	Riboflavin	*11,837*	682	*5812*	966
10	C_13_H_15_NO_5_	265.0955	− 5.7	3.70	*N*-Phenylacetylglutamic acid	*2307*	119	2457	*6993*
11	C_12_H_21_NO_5_	259.1428	3.2	1.82	*N*-(3-oxooctanoyl)homoserine	*2039*	40	*4461*	4323
12	C_11_H_14_N_2_O_3_S	254.0728	1.0	4.21	Alanyl-α-thiophenylglycine	*1480*	29	–	–
13	C_11_H_16_N_2_O_8_	304.0905	− 0.5	1.02	*N*-Acetylaspartylglutamate (NAAG)	*1546*	–	*1176*	675
14	C_9_H_17_NO_4_	203.1164	3.1	1.05	Acetylcarnitine	1317	*119,400*	–	–
15	C_17_H_33_NO_4_	315.2413	1.2	8.51	Decanoylcarnitine	127	*772*	724	*2177*
16	C_10_H_19_NO_4_	217.1319	2.5	1.40	Propanoylcarnitine	3948	*13,560*	5684	*10,619*
17	C_10_H_19_NO_5_	233.1257	− 2.8	13.90	Hydroxypropionylcarnitine	592	*1839*	*6303*	1398
18	C_11_H_21_NO_5_	247.1425	2.0	1.07	Hydroxybutyrylcarnitine	9841	*22,738*	2036	*11,538*
19	C_7_H_15_NO_3_	161.1055	1.9	0.85	Carnitine	120,165	*194,041*	53,165	*77,358*
20	C_19_H_35_NO_4_	341.2569	1.3	6.62	2-Dodecenoylcarnitine	6219	*8800*	417	*1728*
21	C_18_H_35_NO_4_	329.2570	1.1	9.52	4,8-Dimethylnonanoylcarnitine	*2982*	2875	147	*1106*
22	C_13_H_25_NO_4_	259.1790	2.7	4.66	Hexanoylcarnitine	*4642*	2592	–	–
23	C_13_H_23_NO_6_	289.1527	0.6	1.92	3-Methylglutarylcarnitine	*1888*	328	4766	*16,400*
24	C_11_H_19_NO_4_	229.1319	2.1	1.67	Butenylcarnitine	–	–	1109	*3383*
25	C_14_H_27_NO_4_	273.1947	2.4	5.36	Heptanoylcarnitine	–	–	1734	*3177*
26	C_16_H_31_NO_4_	301.2259	1.9	7.17	2,6-Dimethylheptanoylcarnitine	–	–	14,606	*59,515*
27	C_14_H_25_NO_6_	303.1685	1.1	2.78	Pimelylcarnitine	–	–	2036	*13,598*
28	C_19_H_35_NO_6_	373.2459	− 1.3	6.78	Dodecanedioylcarnitine	–	–	294	*2114*

For each pair (tissue, normal or cancer and urine, control or cancer), the high abundance values are set in italics. Putative identifications are given if mentioned in the text, if they are known human metabolites or otherwise may seem relevant to include

“–” peak not detected

^a^Experimental monoisotopic neutral mass

### Analysis of water extracts of tissue

A total of 948 features were detected in the set. The relative similarities and differences between the metabolomes of the different samples were studied using statistical methods. Principal component analysis (PCA) did not yield a clear separation between cancer and normal tissue. Therefore, supervised statistics, i.e., partial least square discriminant analysis (PLS-DA), was applied as it enhances the separation between groups by rotating the PCA components.

**Fig. 1:**
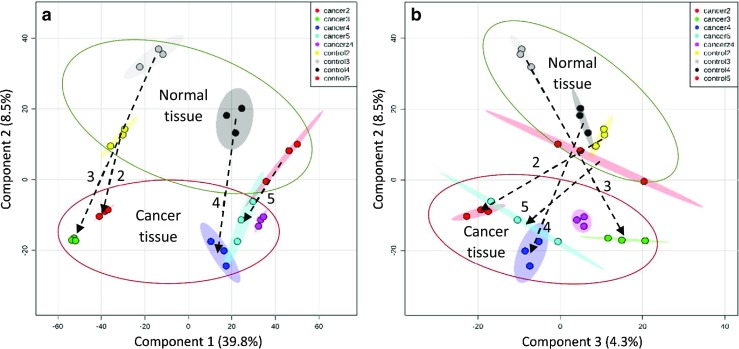
PLS-DA plot based on LC-MS data processed in Metaboanalyst 3.0, showing **a** component 2 versus component 1 and **b** component 2 versus component 3. For each patient, an arrow starts at the location of the normal tissue and ends at the location of the respective cancer tissue. Sample “cancerz4” is based on an inferior vena cava tumor thrombus tissue

Figure 1a shows the results for component 1 (C1) versus component 2 (C2) and Fig. 1b for C2 versus C3. For each patient, an arrow originates at the normal tissue and ends at the cancer tissue, thus representing the metabolomic changes caused by a transition to cancerous growth.

It is seen that it is mainly C2 that separates cancer from normal tissues, while C1 is associated mainly with those interpatient differences that are not strongly dependent on the development of renal cancer. In the present sample set, C1 mainly separates out patients 2 and 3 from patients 4 and 5. It is seen that C2 achieves a clear separation between normal and cancerous renal tissue. (The separation for patient 5 is less clear, due to unexplained outliers in each triplicate experiment).

It is noted that the respective cancer tissues have, for all four patients, a somewhat more negative value for C1. Based on this observation, it would seem that the metabolome differentially associated with the development of renal cancer has a pattern in common with interpatient differences for normal tissue. Expressed differently, the normal tissue metabolisms of some patients are, in some respects, more “cancer like” than those of other patients.

As seen in Fig. 1, component 2 separates patients, based on the metabolic changes that occur upon cancer development. While such difference may also be related to a differential response to influences external to the kidney, such as diet, clues to possible metabolic patterns useful for the classification of renal cancers are likely to be found within C3 and in the magnitude of the change in C2.

In conclusion, it is demonstrated that with PLS-DA of metabolomic data, it is possible to discriminate between cancerous and normal renal tissue [47]. “While the authors acknowledge that this conclusion is based on analysis of data obtained from a small sample pool, a much larger investigation that involves over 100 patients is currently in progress to confirm differences, reported in the pilot study herein, between metabolomic profiles of cancer and healthy renal tissues.

**Fig. 2:**
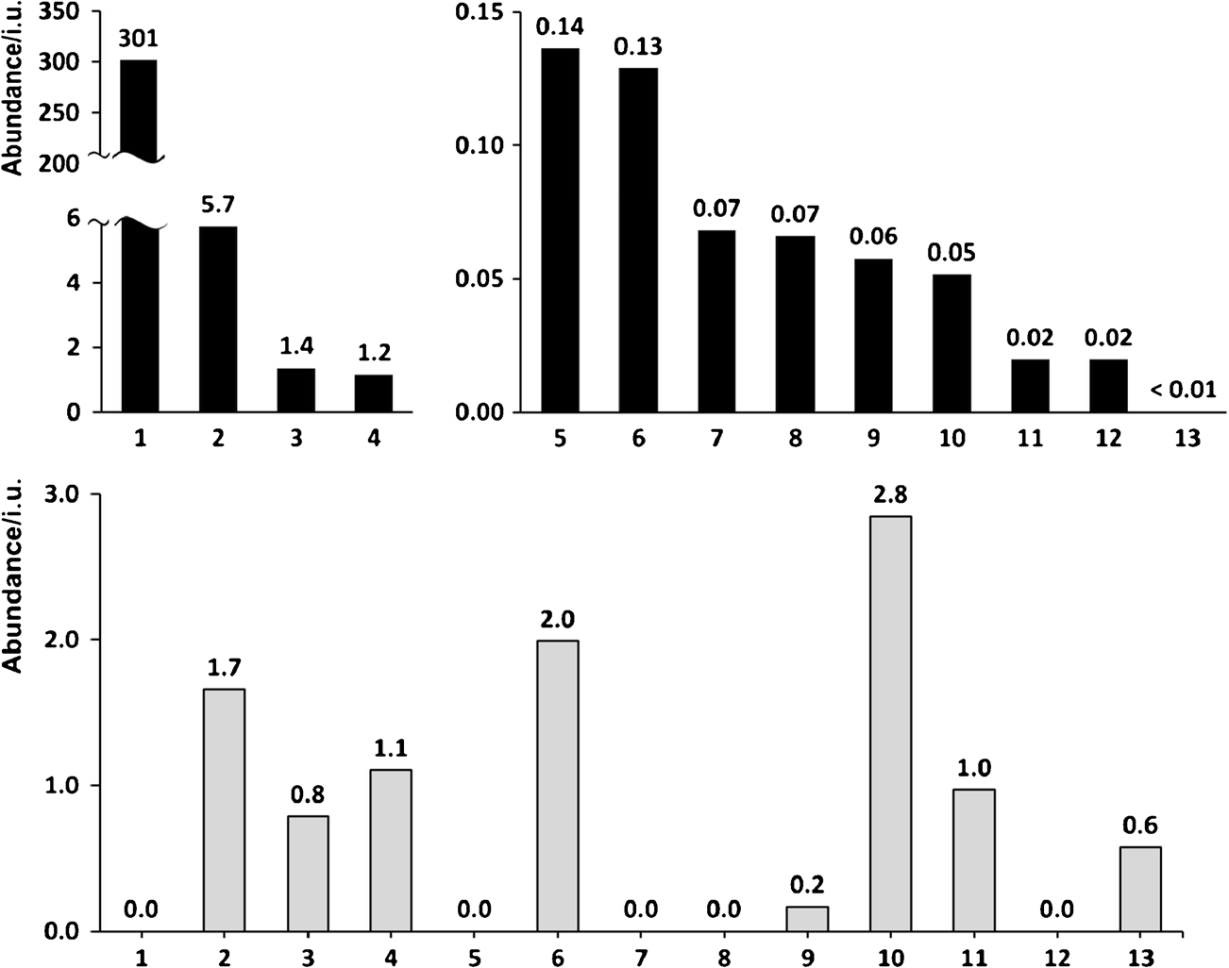
Cancer-to-control abundance ratios for features **1**–**13** found in tissue (black bars, top) and urine (gray bars, bottom)

**Fig. 3:**
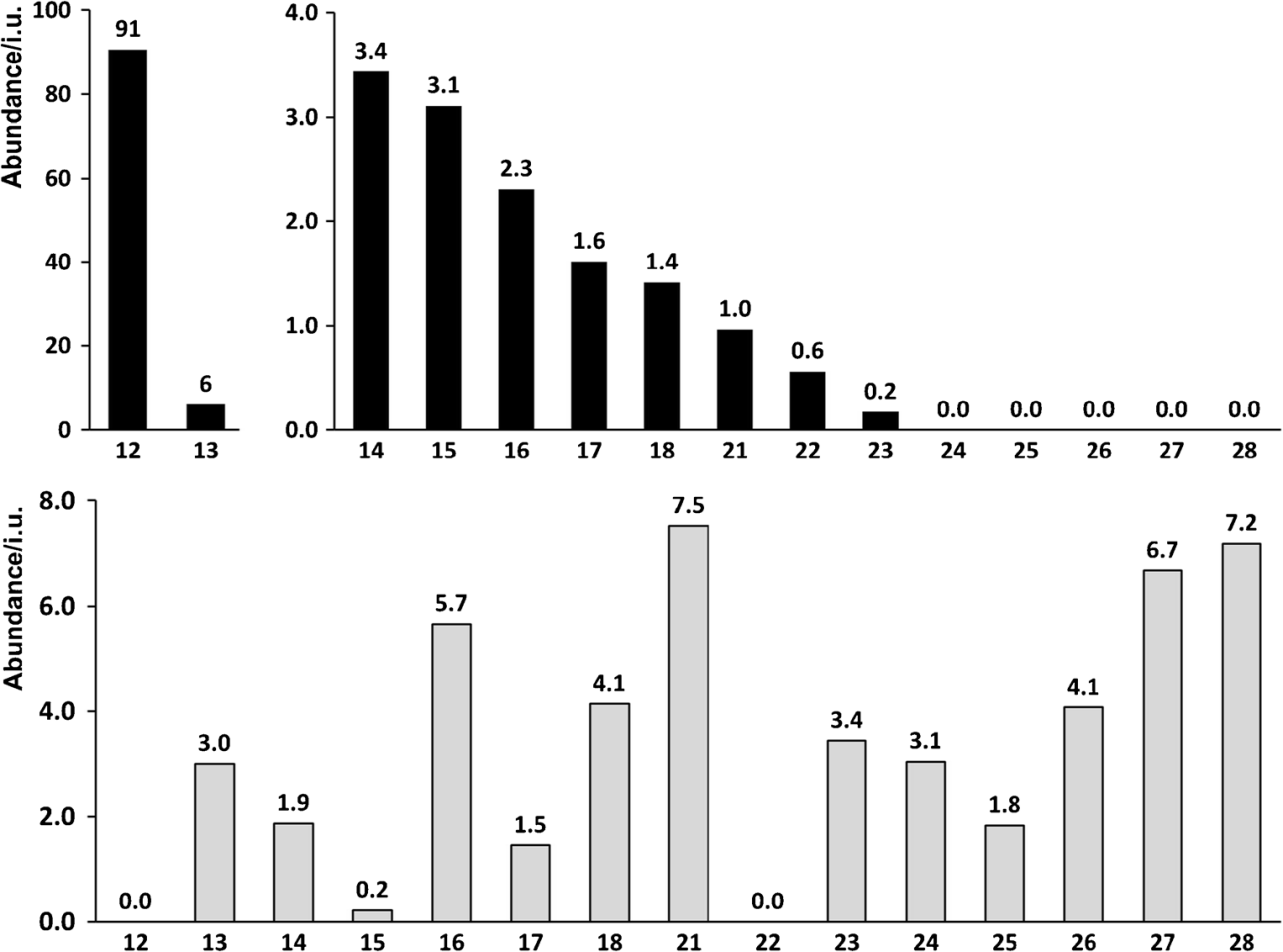
Cancer-to-control abundance ratios for features **12**–**28** found in tissue (black bars, top) and urine (gray bars, bottom)

Of the 948 features detected in the tissue samples, the abundancies for a large majority were not significantly different in cancer versus normal tissue. Using a minimum fold change of 2, seven features were found to have a higher average abundance in the cancer tissue and nine compounds a higher average abundance in the normal tissue. These are listed in Table 2 as compounds **1**, **2**, **12–16**, **6**, **10**, **13**, **16**, **18**, **23**, **24**, and **26–28**, respectively. A listing of compounds with standard errors is in Tables S1 and S2 in the ESM. The cancer-to-control abundance ratios for these features are shown in the form of bar charts in Figs. 2 and 3.

Two features that were over-abundant in cancer tissue were putatively identified as carnitines (**14**, **15**). In particular, acetylcarnitine (**14**) had a very high abundance in cancer tissue, while the abundance in normal renal tissue was two orders of magnitude lower. Decanoylcarnitine (**15**), was also over-expressed in the cancer tissue, though at much lower abundance. Similar observations have been made previously [23, 24] and attributed to fatty acid oxidation disorders (FAOD) and inhibition of the β-oxidation pathway. Several additional carnitines were putatively identified in this work (**14–28**). Five of these (**16–20**) were over-expressed in cancer tissue, though they did not fulfill minimum fold change requirements. Acetylcarnitine, is an acetic acid ester of carnitine, which for example is used by the body to transport fatty acids into the matrices of mammalian mitochondria where fatty acid metabolism occurs [48]. Acetylcarnitine is naturally found in healthy human body, but it is also taken as a dietary supplement. In human plasma and tissues, acetylcarnitine is the most abundant naturally occurring derivative. Acetylcarnitine was recently pointed out as a promising biomarker for hepatocellular carcinoma [49].

The abundances of carnitine (**19**) were high both in tissue and urine samples. Cancer-to-control ratios were found to be moderately high being 1.6 and 1.5 for tissue and urine cancer/control pairs, respectively; it should be noted that tissue result is similar to the one shown by Ganti and co-workers [23]. Much more interesting results were found for other carnitines. For example, hydroxyacylcarnitines found such as hydroxybutyrylcarnitine (**18**) and hydroxypropionylcarnitine (**17**) were found in tissue with 2.3 and 3.1 cancer-to-control ratios, respectively. What is interesting, first compound—hydroxybutyrylcarnitine was also found to be even in higher abundance in cancer urine with a ratio of 5.7; result similar to the one shown by Ganti et al. who showed also higher cancer patients’ concentration of this compound (ca. four times) in urine comparison. Two other carnitines were found to show similar pattern of ratios—4,8-dimethylnonanoylcarnitine (**21**) and 2-dodecenoylcarnitine (**20**) for which cancer-to-control ratios grow from 1.0 to 7.5 in case of **21** and from 1.4 to 4.1 for **20** when switching from tissue to urine results. Two other carnitines—hexanoylcarnitine (**22**) and 3-methylglutarylcarnitine (**23**) present higher abundances in normal tissue samples, and only **23** was detected in urine with 3.4 cancer-to-control ratio.

Other carnitines found exclusively in urine samples such as butenylcarnitine (**24**), heptanoylcarnitine (**25**), 2,6-dimethylheptanoylcarnitine (**26**), pimelylcarnitine (**27**), and dodecanedioylcarnitine (**28**) were detected exclusively in urine, all of them were in higher abundances in cancer patient urine with ratios of 3.1, 1.8, 4.1, 6.7, and 7.2 for compounds **24–28**, respectively. It should be noted that **26** was also found by Ganti et al. with a cancer-to-control ratio of 2.0.

The abundance of feature **1** (Table 2) appeared to be about 300 times higher in the tumor tissue extracts than in the normal tissue control samples. Using available databases, this compound was putatively assigned to difructose anhydride, a non-digestible disaccharide which stimulates calcium absorption in rat and human intestine [50]. This compound is not included in the HMDB and does not seem to have been implicated in cancer metabolism. It is, however, known that cancer tissue often contains oxidated sugars and lipids [51, 52].

Feature **2** is putatively identified with norepinephrine sulfate, which is known to be present in both kidneys and in urine. Norepinephrine sulfate is related to epinephrine, which is an important hormone and neurotransmitter that is involved in a multitude of metabolic pathways. In particular, epinephrine is known to protect cancer cells from apoptosis [53].

Both features **1** and **2** are over-abundant not only in cancer tissue but also in urine samples from patients with kidney cancer. They are discussed further below under “Cross-comparison of tissue and urine results.”

As mentioned above, 13 features were found to have a higher abundance in normal tissue, as compared wwith cancer tissue, features **5–13** in Table 2. One of the thirteen features was not detected at all in cancer tissue, namely feature **13**. Feature **13** is putatively identified with *N*-acetyl-aspartyl-glutamate (NAAG).

The biggest fold changes, > 50, for normal versus cancer tissue were observed for features **11** and **12**. Feature **11**, but not **12,** was present also in urine but without significant difference between RCC and control patient groups. Feature **11** is putatively identified as *N*-(3-oxooctanoyl)homoserine, and feature **12** as alanyl-α-thiophenylglycine or *S*-(phenylacetothiohydroximoyl)-cysteine. The latter compound is involved in the biosynthesis of glucosinolates from phenylalanine.

Two features that were over-abundant in normal tissue, were also significantly more abundant in normal versus cancer urine. Apart from **13** (NAAG), this was the case also for **9,** which has the putative identification of riboflavin. Both will be discussed in more detail below.

For several of the features that were over-abundant in normal tissue, it was observed that the abundances in the urine samples were reversed, i.e., higher in cancer than in normal urine, albeit with a smaller fold change. This was the case for features **6**, **10**, and **23**. This pattern can be expected for compounds that are not retained or consumed, by cancer tissue, but excreted. Three of the four features have putative identifications based on mass alone but without additional evidence they remain highly uncertain.

Five of the features that were over-abundant in normal tissue were not detected in urine, namely **7**, **5**, **8**, **12**, **15**, and **75**. Feature **8** was 15-fold over-abundant in normal as compared with cancer tissue. Assignment to a compound was difficult as metabolite data base searches yielded 18 isomers (Table S1 in the ESM). Feature **7**, also over-abundant by 15-fold in normal tissue, was putatively identified with aminononanoic acid, an amino fatty acid.

### Analysis of THF extracts of tissue

Additional analysis based on THF-extracts of metabolites was performed. Selected results of this analysis are presented in Table 3. All compounds presented in Table 3 were also found in water-extract-based analyses. The majority of compounds show the same qualitative trends of the cancer-to-normal abundance ratios as for water-extract analysis discussed in “Analysis of water extracts of tissue”. A ratio higher than six was found for features **1**, **3**, **14,** and **29**. Of there, feature **3**—glutamine, was found with the exceptionally high cancer-to-normal ratio of 65.6. In the water-based analysis, the ratio was close to one. In conclusion, acetylcarnitine (**14**) is shown to be an excellent candidate for a tissue-based biomarker in both water and THF extracts; unfortunately, it was not found in urine samples (vide infra).

**Table 3:** List of features that are over-abundant in either cancer tissue or normal tissue extracted with THF

No.	Formula	Mass^a^	Mass error (ppm)	Retention time (min)	Putative metabolite	Average abundances		Cancer/normal ratio
						Normal	Cancer	
1	C_12_H_20_O_10_	324.1060	1.2	0.81	Bis-d-fructose 2′,1:2,1′-dianhydride	137	2221	*16.2*
3	C_5_H_10_N_2_O_3_	146.0694	1.7	0.80	Glutamine	197	12,938	*65.6*
14	C_9_H_17_NO_4_	203.1161	1.9	0.90	Acetylcarnitine	11,150	151,110	*13.6*
15	C_17_H_33_NO_4_	315.2406	− 1.1	8.45	Decanoylcarnitine	446	2071	4.6
16	C_10_H_19_NO_4_	217.1320	2.6	0.90	Propanoylcarnitine	2569	10,807	4.2
19	C_7_H_15_NO_3_	161.1054	1.4	0.86	Carnitine	19,208	76,516	4.0
20	C_19_H_35_NO_4_	341.2571	1.6	9.11	2-Dodecenoylcarnitine	317	762	2.4
21	C_18_H_35_NO_4_	329.2570	1.1	9.46	4,8-Dimethylnonanoylcarnitine	6005	5657	0.9
22	C_13_H_25_NO_4_	259.1789	2.0	4.59	Hexanoylcarnitine	881	2258	2.6
25	C_14_H_27_NO_4_	273.1938	− 0.9	0.90	Heptanoylcarnitine	1522	874	0.6
29	C_40_H_52_O_2_	564.3962	− 1.0	17.09	Unidentified cancer tissue biomarker	64	738	*11.6*

Cancer/normal ratios higher than six are set in italics

^a^Experimental monoisotopic neutral mass

### Analysis of urine samples

Among the 3368 features detected in the set of urine samples, 16 were determined to be over-abundant (features **2**, **4**, **6**, **10**, **15**, **16**, **18–21**, **23–28**) and 44 to be under-abundant (features **3**, **9**, **11**, **13**, **17**) in cancer urine, when compared with control urine. The abundance data is presented as bar charts in Figs. 2 and 3.

The abundance pattern for the 11 features (**6**, **10**, **15**, **18**, **20**, **21**, **23**, **24**, **26**–**28**) over-abundant in cancer urine have a dominant common pattern. All except features **24–28** were detected also in tissue. Seven of the features were either not detected at all, or detected with an insignificant abundance, in urine from non-cancer patients. Third, the hits in the MMDB and HMDB were few, and none were obvious candidates for known human metabolites. While other scenarios are possible, this pattern is consistent with the features being due to drugs given to the cancer patients or to their metabolites. Such compounds are indeed expected to be present in both types of tissue and in cancer urine, but not in control urine. Because a wide range of synthetic compounds are possible, any putative identifications would be very unreliable and not included in Table 2.

### Cross-comparison of tissue and urine results

It is preferable for a cancer biomarker to be detected in an easily available body fluid, preferably urine. The present study involves four different types of samples. The paired cancer and normal tissues are both obtained from patients with cancer, while the cancer urine samples were obtained from the patients that donated the tissue while the normal urine samples were obtained from a different group of patients that had no signs of renal cancer. As illustrated in the previous sections, comparing the abundances of features between these four types of samples, give important clues as to the origin of compounds and, therefore, to the identification of possible renal cancer biomarkers.

The most obviously promising biomarkers would be compounds that are present in cancer cells and leaked into the urinary space [35]. Unless such compounds also diffuse into neighboring normal renal tissue, they should be found among features that are over-abundant in both renal cancer tissue and in the urine of cancer patients, but essentially absent in normal tissue and control urine. In the present study, seven features stand out in this respect: **2**, **4**, **15**, **16**, and **18**–**20**. The charts in Figs. 2 and 3 graphically illustrates the abundance ratios of these features.

Compounds that are under-expressed, or absent, in cancer tissue, as opposed to healthy tissue are also potential biomarkers. However, such compounds may, or may not, be under-expressed in cancer urine as they are likely to still enter the urine from healthy kidney tissue. Notable features that are under-abundant in both cancer tissue and in cancer urine are **9**, **11**, and **13**.

Feature **13** at *m/z* 304.0905 yielded one hit in MMCD, namely *N*-acetylaspartylglutamate (NAAG). This feature was only detected in normal tissue and not in cancer tissue. While NAAG is normally present in urine of healthy subjects, it was here found to be less abundant in urine from the cancer patients. NAAG is one of the three most prevalent dipeptide neurotransmitters in the mammalian nervous system. The simplest explanation for the absence of NAAG in renal cancer may be denervation of the tumor, something that is commonly observed and has been reported to enhance cancer metastasis [54]. However, NAAG appears to play important roles in the neural system related to regulation of energy supply [55, 56], and it cannot be excluded that its absence is essential for tumor growth.

NAAG in urine does not seem to previously have been considered for cancer detection. However, the observation that the decrease in NAAG in the urine of cancer patients is substantial, suggests that the concentration of NAAG is urine may be used as an indicator of RCC size or activity.

Feature **9** is also observed to be over-abundant in both cancer tissue and urine. This feature was putatively assigned to riboflavin. This compound is one of eight B-complex vitamins, and it plays a key role in maintaining human health. The significance of riboflavin was discussed in many publications [57, 58].

Features that are over-abundant in both cancer tissue and cancer urine (**2**, **4**, **15**, **16**, **18**–**20**) are, as mentioned above, strong candidates for potential kidney cancer biomarkers. Feature **15** is putatively identified with decanoylcarnitine, which is a carnitine ester with decanoic acid. This compound is present in blood plasma in cases of fatty acid oxidation defects (FAOD), such as long-chain 3-hydroxylacyl-CoA dehydrogenase (LCHAD) deficiency, carnitine palmitoyltransferase I (CPT I) deficiency, carnitine palmitoyltransferase II (CPT II) deficiency, and medium-chain acyl-coenzyme A dehydrogenase deficiency [59].

It is of high importance to state that that this compound was also found in urine samples from cancer patients at three times higher abundance compared with control (vide infra). Thus, it is a very good candidate for a new kidney cancer biomarker. Acylcarnitines, which are intermediates in the key energy metabolic pathways of fatty acid β-oxidation and amino acid catabolism, were found previously at higher concentrations in tumor tissues [23, 24] and urine as compared with a set of matched control patients without RCC. Additionally, some studies have reported that several carnitine-type metabolites could also be considered early RCC biomarkers. Acylcarnitines could be emanating either from the tumor itself, or their appearance is the result of a systemic response to the presence of the tumor cells. A possible explanation for these changes is that highly undifferentiated cancer cells require more energy; they rely on fatty acid β-oxidation to maintain its viability. On the other hand, enzymes of β-oxidation seem to be downregulated as RCC progresses, suggesting reduced oxidation of acyl-CoAs and, consequently, accumulation of carnitine species in cancer cells [24].

Norepinephrine sulfate (NE sulfate) showed significant increases both in cancer tissue and urine samples from patients with kidney cancer (**2**, Fig. 2). This compound was identified at concentrations approximately 5-fold higher in RCC tissue relative to normal but also at about two times higher concentrations in urine samples from the patients with kidney cancer compared with healthy control group (**2**, Fig. 2). Norepinephrine sulfate is formed from free norepinephrine by the enzyme phenol sulfotransferase. In the human body, norepinephrine sulfate is present in plasma in concentration about two to four times higher than free norepinephrine [60]. NE sulfate concentration in plasma increases after sympathetic nervous system activation by an exhausting incremental exercise test and remain elevated up to 2 h after exercise.

## Conclusions

Liquid chromatography/high-resolution mass spectrometry analysis of extracts from cancer and healthy tissue regions allowed the identification of up- and downregulated compounds that could potentially serve as renal cancer biomarkers, ccRCC. Similar analyses of urine from cancer patients and from a healthy control group yielded additional putative biomarkers. The putative identifications of compounds were based on exact mass and on data base hits on important human metabolites, known to be relevant for cancer. Cross-comparison of two sets of results allowed the identification of four kidney cancer biomarkers that are either over- or under-expressed in both cancer tissue and urine from cancer patients. Hydroxybutyrylcarnitine, decanoylcarnitine, propanoylcarnitine, carnitine, dodecanoylcarnitine, and norepinephrine sulfate were found in distinctly higher concentrations in both cancer tissues and in urine of cancer patients compared with controls. In contrast, feature assigned to riboflavin and NAAG were present at significantly higher concentrations both in normal kidney tissue as compared with renal cancer tissue and in urine samples of healthy persons than in urine from the cancer patients. All eight mentioned compounds may be considered potential clear cell renal carcinoma biomarkers. Preliminary research presented in this work will be followed in the future with larger-scale study based on higher amount of patients.

## Electronic supplementary material

ESM 1 (PDF 590 kb)
