# Supplementary material for: Metabolomic study of human tissue and urine in clear cell renal carcinoma by LC-HRMS and PLS-DA
Source: Anal Bioanal Chem. 2018 Apr 16;410(16):3859–69. doi: 10.1007/s00216-018-1059-x (PMC5956006; doi:10.1007/s00216-018-1059-x)
Supplement: Supplementary file 1 — (PDF 590 kb) [file 216_2018_1059_MOESM1_ESM.pdf]

Analytical and Bioanalytical Chemistry

Electronic Supplementary Material

Metabolomic study of human tissue and urine in clear cell renal carcinoma by LC-HRMS and PLS-DA

Joanna Nizioł, Vincent Bonifay, Krzysztof Ossoliński, Tadeusz Ossoliński, Anna Ossolińska, Jan Sunner, Iwona Beech, Adrian Arendowski, Tomasz Ruman

Content

Table S1 List of putatively identified metabolites in tissue and urine samples

Table S2 List of putatively identified metabolites in tissue samples – analysis of THF extracts

Fig. S1 Examples of total ion chromatograms for tissue extracts (red/top – cancer tissue, green/bottom – normal tissue)

Fig. S2 Examples of total ion chromatograms for urine samples (red/top – cancer patient urine, green/bottom – control urine)

Table S1 List of putatively identified metabolites in tissue and urine samples – analysis of water extracts

No.	Formula	Mass[1]	Putative metabolite[2]	Average abundances[3]							
				Tissue				Urine			
				Normal	Normal SD	Cancer	Cancer SD	Control	Control SD	Cancer	Cancer SD
1	$C_{12}H_{20}O_{10}$	324.1059	Bis-fructose 2',1:2,1'-dianhydride	**116**	88	**34973**	24360	**-**		**-**	
2	$C_8H_{11}NO_6S$	249.0309	Norepinephrine sulfate	**174**	301	**999**	1285	**380**	66	**630**	670
3	$C_5H_{10}N_2O_3$	146.0694	Glutamine	**23471**	20890	**31944**	17532	**15542**	7910	**12212**	8638
4	$C_5H_9NO_4$	147.0532	Glutamate	**170025**	107354	**196812**	150964	**1451**	324	**1603**	637
5	$C_{10}H_{21}NOS$	203.1335	Methylthiononanaldoxime	**46617**	21626	**6349**	2664	**-**	-	**-**	-
6	$C_9H_{17}NO_3$	187.1156	*N*-Heptanoylglycine	**8801**	8973	**1133**	1053	**513**	301	**1021**	1443
7	$C_9H_{19}NO_2$	173.1418	Amino-nonanoic acid	**6276**	2727	**427**	65	**-**		**-**	
8	$C_{33}H_{40}O_{22}$	788.2030	Quercetin sophoroside glucoside	**3509**	3628	**231**	199	**-**	-	**-**	-
9	$C_{17}H_{20}N_4O_6$	376.1384	Riboflavin	**11837**	11164	**682**	269	**5812**	3573	**966**	1246
10	$C_{13}H_{15}NO_5$	265.0955	*N*-Phenylacetylglutamic acid	**2307**	905	**119**	165	**2457**	1115	**6993**	9261
11	$C_{12}H_{21}NO_5$	259.1428	*N*-(3-oxooctanoyl)homoserine	**2039**	2555	**40**	69	**4461**	2744	**4323**	5812
12	$C_{11}H_{14}N_2O_3S$	254.0728	Alanyl-α-thiophenylglycine	**1480**	764	**29**	50	**-**	-	**-**	-
13	$C_{11}H_{16}N_2O_8$	304.0905	*N*-Acetylaspartylglutamate (NAAG)	**1546**	968	**-**	-	**1176**	966	**675**	687
14	$C_9H_{17}NO_4$	203.1164	Acetylcarnitine	**1317**	403	**119400**	154065	**-**		**-**	
15	$C_{17}H_{33}NO_4$	315.2413	Decanoylcarnitine	**127**	167	**772**	288	**724**	167	**2177**	3055
16	$C_{10}H_{19}NO_4$	217.1319	Propanoylcarnitine	**3948**	1919	**13560**	7478	**5684**	1187	**10619**	13054
17	$C_{10}H_{19}NO_5$	233.1257	Hydroxypropionylcarnitine	**592**	681	**1839**	1593	**6303**	3475	**1398**	652

**18**	$C_{11}H_{21}NO_5$	247.1425	Hydroxybutyrylcarnitine	**9841**	2726	**22738**	18525	**2036**	464	**11538**	19962
**19**	$C_7H_{15}NO_3$	161.1055	Carnitine	**120165**	82739	**194041**	108019	**53165**	23006	**77358**	85980
**20**	$C_{19}H_{35}NO_4$	341.2569	2-Dodecenoylcarnitine	**6219**	9604	**8800**	12269	**417**	59	**1728**	2769
**21**	$C_{18}H_{35}NO_4$	329.2570	4,8-Dimethylnonanoylcarnitine	**2982**	515	**2875**	355	**147**	64	**1106**	1985
**22**	$C_{13}H_{25}NO_4$	259.1790	Hexanoylcarnitine	**4642**	2445	**2592**	1381	**-**	-	**-**	-
**23**	$C_{13}H_{23}NO_6$	289.1527	3-Methylglutarylcarnitine	**1888**	1100	**328**	55	**4766**	1934	**16400**	15177
**24**	$C_{11}H_{19}NO_4$	229.1319	Butenylcarnitine	**-**	-	**-**	-	**1109**	485	**3383**	4212
**25**	$C_{14}H_{27}NO_4$	273.1947	Heptanoylcarnitine	**-**	-	**-**	-	**1734**	1	**3177**	3164
**26**	$C_{16}H_{31}NO_4$	301.2259	2,6-Dimethylheptanoylcarnitine	**-**	-	**-**	-	**14606**	2510	**59515**	85917
**27**	$C_{14}H_{25}NO_6$	303.1685	Pimelylcarnitine	**-**	-	**-**	-	**2036**	921	**13598**	19339
**28**	$C_{19}H_{35}NO_6$	373.2459	Dodecanedioylcarnitine	**-**	-	**-**	-	**294**	110	**2114**	3573

[1] Experimental monoisotopic neutral mass; [2]putative identification of extracted features with metabolites and other compounds in the IDEOM database; [3]average abundances from all samples of the same type; "-" - peak not detected; PG – glycerophosphoglycerols; PIP – phosphatidylinositol phosphates; PI – phosphatidylinositols; PC – phosphocholines; MG – monoacylglyceride; TG – triacylglyceride; PA – phosphatidylglycerols; PE – glycerophosphoethanolamines; SP – sphingosines; PS – phosphatidylserines

Table S2 List of putatively identified metabolites in tissue samples – analysis of THF extracts

No.	Formula	Mass[1]	Putative metabolite[2]	Average abundances[3]			
				Tissue			
				Normal	Normal SD	Cancer	Cancer SD
**1**	$C_{12}H_{20}O_{10}$	324.1060	Bis-D-fructose 2',1:2,1'-dianhydride	**137**	173	**2221**	2047
**3**	$C_5H_{10}N_2O_3$	146.0694	Glutamine	**197**	202	**12938**	16883
**14**	$C_9H_{17}NO_4$	203.1161	Acetylcarnitine	**11150**	15016	**151110**	139733
**15**	$C_{17}H_{33}NO_4$	315.2406	Decanoylcarnitine	**446**	232	**2071**	2157
**16**	$C_{10}H_{19}NO_4$	217.1320	Propanoylcarnitine	**2569**	3353	**10807**	14085
**19**	$C_7H_{15}NO_3$	161.1054	Carnitine	**19208**	22821	**76516**	69447
**20**	$C_{19}H_{35}NO_4$	341.2571	2-Dodecenoylcarnitine	**317**	212	**762**	514
**21**	$C_{18}H_{35}NO_4$	329.2570	4,8-Dimethylnonanoylcarnitine	**6005**	1251	**5657**	1463
**22**	$C_{13}H_{25}NO_4$	259.1789	Hexanoylcarnitine	**881**	752	**2258**	2281
**25**	$C_{14}H_{27}NO_4$	273.1938	Heptanoylcarnitine	**1522**	1441	**874**	985
**29**	$C_{40}H_{52}O_2$	564.3962	Unidentified cancer tissue biomarker	**64**	64	**738**	712

[1] Experimental monoisotopic neutral mass; [2]putative identification of extracted features with metabolites and other compounds in the IDEOM database; [3]average abundances from all samples of the same type

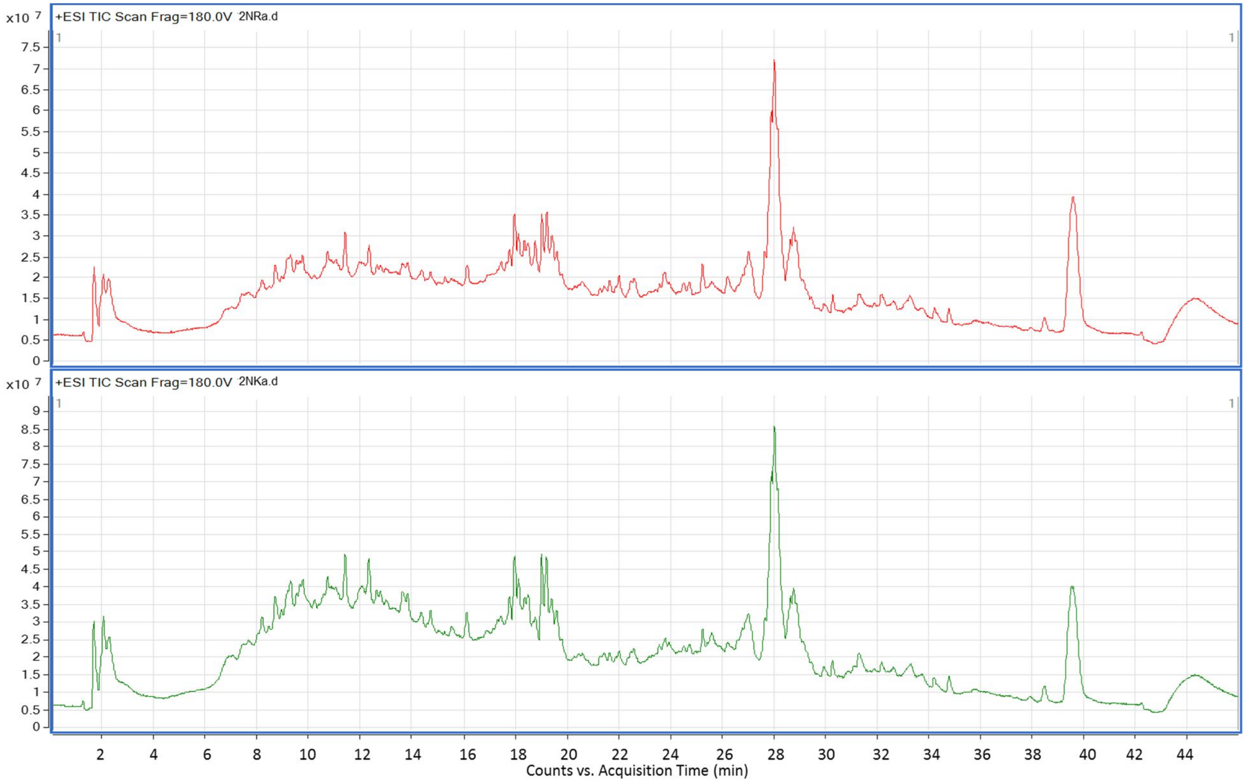

Fig. S1 Examples of total ion chromatograms for tissue extracts (red/top – cancer tissue, green/bottom – normal tissue)

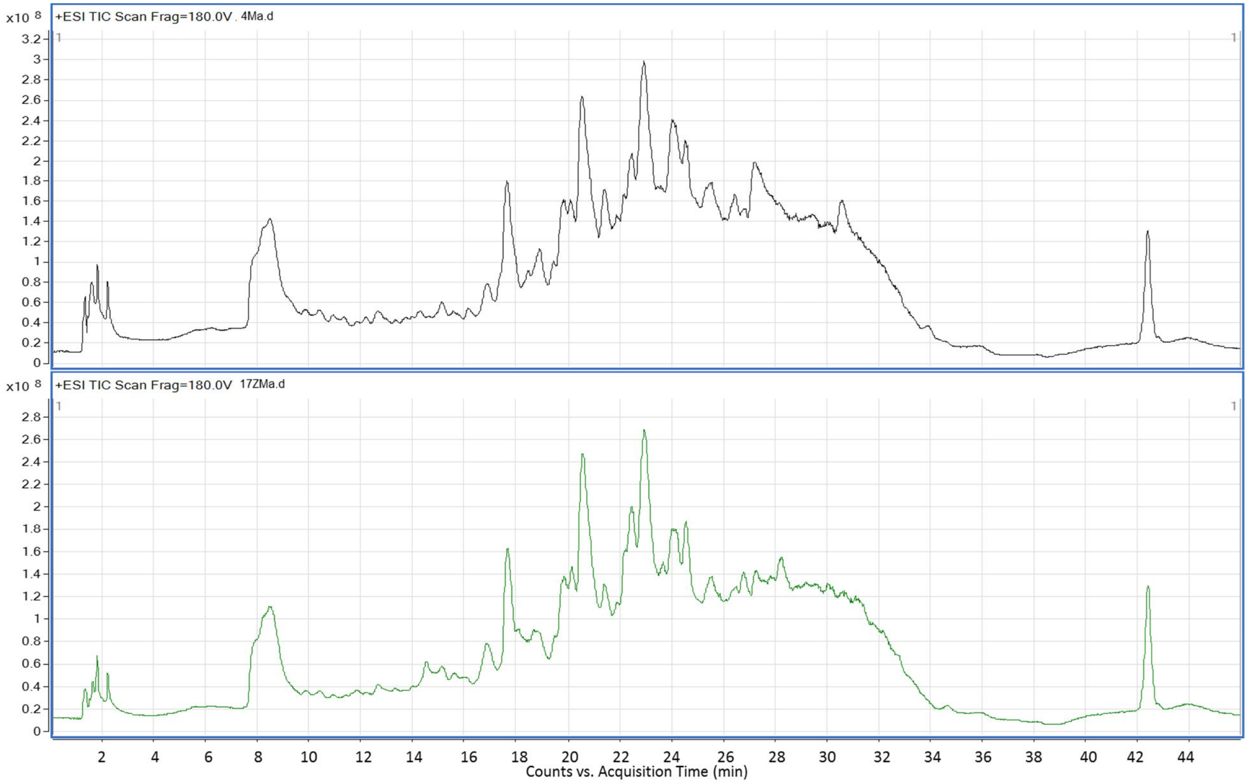

Fig. S2 Examples of total ion chromatograms for urine samples (red/top – cancer patient urine, green/bottom – control urine)
